# Supplementary material for: Protective Effect of Leisure-Time Physical Activity and Resistance Training on Nonalcoholic Fatty Liver Disease: A Nationwide Cross-Sectional Study
Source: Int J Environ Res Public Health. 2022 Feb 18;19(4):2350. doi: 10.3390/ijerph19042350 (PMC8872481; doi:10.3390/ijerph19042350)
Supplement: Supplementary file 1 [file ijerph-19-02350-s001.zip › ijerph-1592416-supplementary.pdf]

Table S1. Odds ratios for NAFLD based on the amount of leisure-time PA per week and sex.

	**N**	**NAFLD** (%)	**PA-time**	**Model 1** OR (95% CI)	**Model 2** OR (95% CI)	**Model 3** OR (95% CI)
**Total**	137,555	18.41				
Non-PA	68,929	19.15	0.00	1 (reference)[b]	1 (reference)[b]	1 (reference)[b]
Low-PA (< 150 min)	17,746	18.07	90.46 ± 32.29	0.92 (0.88–0.96)***	1.01 (0.96–1.06)	0.97 (0.92–1.04)
Mid-PA (150–299 min)	22,053	17.18	211.01 ± 30.33	0.83 (0.80–0.87)****	0.91 (0.87–0.96)***	0.85 (0.81–0.90)****
High-PA (≥ 300 min)	28,827	17.79	500.61 ± 246.20	0.79 (0.77–0.82)****	0.86 (0.82–0.90)****	0.76 (0.72–0.80)****
**Men**	40,607	30.54				
Non-PA	18,597	31.41	0.00	1 (reference)[b]	1 (reference)[b]	1 (reference)[b]
Low-PA (< 150 min)	5642	31.04	88.69 ± 32.28	0.98 (0.92–1.05)	0.96 (0.89–1.03)	0.93 (0.84–1.01)
Mid-PA (150–299 min)	6813	29.96	212.22 ± 33.22	0.93 (0.88–0.99)*	0.88 (0.82–0.94)***	0.84 (0.77–0.91)****
High-PA (≥ 300 min)	9555	28.95	529.94 ± 279.79	0.89 (0.84–0.94)****	0.81 (0.76–0.86)****	0.75 (0.70–0.81)****
**Women**	96,948	13.33				
Non-PA	50,332	14.62	0.00	1 (reference)[b]	1 (reference)[a]	1 (reference)[b]
Low-PA (< 150 min)	12,104	12.02	91.29 ± 32.27	0.84 (0.79–0.89)****	0.99 (0.92–1.07)	0.98 (0.90–1.07)
Mid-PA (150–299 min)	15,240	11.47	210.47 ± 28.93	0.76 (0.71–0.80)****	0.91 (0.85–0.97)**	0.86 (0.79–0.93)****
High-PA (≥ 300 min)	19,272	12.25	486.07 ± 226.33	0.78 (0.74–0.82)****	0.92 (0.87–0.98)**	0.80 (0.75–0.86)****

NAFLD, nonalcoholic fatty liver disease; PA, physical activity; PA-time, a total time participating regularly in any sports or exercise to the point of sweating; OR, odds ratio; CI, confidence interval; BMI, body mass index; WC, waist circumference; T-Chol, total cholesterol; ALT, alanine aminotransferase; *, $p < 0.05$; **, $p < 0.01$; ***, $p < 0.001$; ****, $p < 0.0001$; [a], $p < 0.01$ for the test for trend of ORs; [b], $p < 0.0001$ for the test for trend of ORs. Model 1 was adjusted for age and sex; Model 2 was adjusted for the variables in Model 1, plus drinking, smoking, education level, BMI, and WC; and Model 3 was adjusted for the variables in Model 2, plus T-Chol, ALT, hypertension, and diabetes mellitus.

Table S2. Characteristics of study participants based on leisure-time PA levels and regularity of RT.

Variables	**Low-PA** (n = 86,675)		p-value	**High-PA** (n = 50,880)		p-value
	**Low-PA** (n = 82,588)	**Low-PA+RT** (n = 4087)		**Low-PA** (n = 40,362)	**High-PA** (n = 10,518)	
**Women**, n (%)	60,349 (73.07)	2087 (51.06)	< 0.0001	28,170 (69.79)	6342 (60.30)	< 0.0001
**Age** (years)	52.78 ± 8.51	50.34 ± 7.80	< 0.0001	54.06 ± 8.08	52.05 ± 7.73	< 0.0001
**Education level**, n (%)			< 0.0001			< 0.0001
≤ Elementary school	18,933 (22.92)	317 (7.76)		6796 (16.84)	871 (8.28)	
Middle/high school	45,892 (55.57)	2132 (52.16)		24,037 (59.55)	6185 (58.80)	
≥ College	17,763 (21.51)	1638 (40.08)		9529 (23.61)	3462 (32.92)	
**Drinking habit**, n (%)			< 0.0001			< 0.0001
Never drinker	47,531 (57.55)	1633 (39.96)		21,887 (54.23)	4757 (45.23)	
Ex-drinker	2917 (3.53)	216 (5.28)		1442 (3.57)	479 (4.55)	
Current drinker	32,140 (38.92)	2238 (54.76)		17,033 (42.20)	5282 (50.22)	
**Smoking habit**, n (%)			< 0.0001			< 0.0001
Never smoker	64,793 (78.46)	2684 (65.67)		31,398 (77.79)	7496 (71.27)	
Ex-smoker	8376 (10.14)	849 (20.77)		5714 (14.16)	2075 (19.73)	
Current smoker	9419 (11.40)	554 (13.56)		3250 (8.05)	947 (9.00)	
**PA-time** (min/week)	16.60 ± 37.54	57.45 ± 52.18	< 0.0001	367.93 ± 235.37	402.54 ± 232.70	< 0.0001
**BMI** (kg/m^2)	23.88 ± 3.02	23.84 ± 2.77	0.33	23.92 ± 2.77	23.87 ± 2.66	0.11
**WC** (cm)	80.79 ± 8.77	81.11 ± 8.47	< 0.05	80.65 ± 8.39	80.31 ± 8.32	< 0.001
**SBP** (mmHg)	121.63 ± 15.63	120.83 ± 15.34	< 0.01	122.65 ± 15.53	121.47 ± 14.95	< 0.0001
**DBP** (mmHg)	75.72 ± 10.06	76.06 ± 10.32	< 0.05	76.15 ± 9.91	75.74 ± 9.70	< 0.001
**T-Chol** (mg/dL)	198.20 ± 35.47	195.82 ± 34.43	< 0.0001	198.75 ± 35.11	197.27 ± 34.52	< 0.001
**HDL-C** (mg/dL)	53.14 ± 12.70	53.56 ± 12.49	< 0.05	54.50 ± 13.03	55.68 ± 13.57	< 0.0001
**TG** (mg/dL)	126.39 ± 87.64	126.20 ± 86.02	0.89	120.83 ± 81.08	116.22 ± 80.93	< 0.0001
**FBG** (mg/dL)	94.08 ± 21.01	93.20 ± 18.28	< 0.01	95.07 ± 20.66	93.79 ± 18.39	< 0.0001
**AST** (IU/L)	23.26 ± 20.51	23.07 ± 9.71	0.26	23.26 ± 10.27	23.44 ± 12.85	0.18
**ALT** (IU/L)	21.98 ± 23.16	22.58 ± 16.61	< 0.05	21.35 ± 15.63	21.54 ± 16.96	0.31
**Hypertension**, n (%)	22,444 (27.18)	992 (24.27)	< 0.0001	12,188 (30.20)	2762 (26.26)	< 0.0001
**DM**, n (%)	6632 (8.03)	289 (7.07)	< 0.05	4103 (10.17)	893 (8.49)	< 0.0001
**FSI**	14.89 ± 16.46	15.13 ± 16.94	0.37	14.56 ± 15.57	13.64 ± 15.09	< 0.0001
**NAFLD**, n (%)	15,621 (18.91)	787 (19.26)	0.59	7230 (17.91)	1686 (16.03)	< 0.0001

PA, physical activity; RT, resistance training; PA-time, a total time participating regularly in any sports or exercise to the point of sweating; BMI, body mass index; WC, waist circumference; SBP, systolic blood pressure; DBP, diastolic blood pressure; T-Chol, total cholesterol; HDL-C, high-density lipoprotein cholesterol; TG, triglycerides; FBG, fasting blood glucose; AST, aspartate aminotransferase; ALT, alanine aminotransferase; DM, diabetes mellitus; FSI, Framingham steatosis index; NAFLD, nonalcoholic fatty liver disease.

Table S3. Odds ratios for NAFLD prevalence according to regularity of RT and sex in participants with high leisure-time PA levels.

	N	RT Levels			Model 1	Model 2	Model 3
		Frequency (days/week)	Training Period (month)	≥1 year (%)	OR (95% CI)	OR (95% CI)	OR (95% CI)
**Total**	50,880						
High-PA	40,362	-	-	-	1 (reference)	1 (reference)	1 (reference)
High-PA+RT	10,518	4.36 ± 1.57	18.59 ± 34.22	81.98	0.84 (0.79–0.89)***	0.86 (0.81–0.93)***	0.87 (0.80–0.94)**
**Men**	16,368						
High-PA	12,192	-	-	-	1 (reference)	1 (reference)	1 (reference)
High-PA+RT	4176	4.44 ± 1.70[a]	21.95 ± 44.04[a]	85.56[a]	0.85 (0.79–0.92)***	0.81 (0.74–0.89)***	0.81 (0.73–0.90)**
**Women**	34,512						
High-PA	28,170	-	-	-	1 (reference)	1 (reference)	1 (reference)
High-PA+RT	6342	4.30 ± 1.48[a]	16.38 ± 25.56[a]	79.63[a]	0.86 (0.78–0.94)*	0.97 (0.87–1.08)	0.98 (0.86–1.12)

NAFLD, nonalcoholic fatty liver disease; RT, resistance training; PA, physical activity; OR, odds ratio; CI, confidence interval; BMI, body mass index; WC, waist circumference; T-Chol, total cholesterol; ALT, alanine aminotransferase; PA-time, a total time participating regularly in any sports or exercise to the point of sweating; *, $p < 0.01$; **, $p < 0.001$; ***, $p < 0.0001$; [a], $p < 0.0001$ compared female with male in High-PA+RT. Model 1 was adjusted for age and sex; Model 2 was adjusted for the variables in Model 1, plus drinking, smoking, education level, BMI, and WC; and Model 3 was adjusted for the variables in Model 2, plus T-Chol, ALT, hypertension, diabetes mellitus, and PA-time.
